# Racial Disparities in Breastfeeding Rates in Patients with Heart Disease

**DOI:** 10.1007/s40615-024-01933-1

**Published:** 2024-03-20

**Authors:** Ayamo G. Oben, Christina T Blanchard, Ashton Robinson, Isabel Girling, Joanna M. Joly, Marc Cribbs, Alan Tita, Brian Casey, Rachel Sinkey

**Affiliations:** 1 University of Alabama at Birmingham Marnix E. Heersink School of Medicine, Birmingham, AL USA; 2 Department of Obstetrics & Gynecology, University of Alabama at Birmingham, 1700 6th Ave South, Ste 10270, Birmingham, AL 35233 USA; 3 Center for Women’s Reproductive Health, University of Alabama at Birmingham, Birmingham, AL USA; 4 Department of Medicine, Division of Cardiovascular Disease, University of Alabama, Birmingham, AL USA

## Abstract

**Objective:**

To evaluate racial disparities in breastfeeding rates in patients with heart disease.

**Study Design:**

Retrospective cohort of pregnant patients with maternal cardiac disease managed by a Cardio-Obstetrics program. Patients self-identifying as Non-Hispanic Black (NHB) and Non-Hispanic White (NHW), who attended ≥ 1 prenatal visit at the Cardio-Obstetrics Program and delivered at the same hospital between March 2015 and June 2019 were included. The primary outcome was breastfeeding rate at discharge from the delivery-associated hospitalization. Secondary outcomes included breastfeeding intent on admission and breastfeeding rates at the postpartum visit among patients who initiated breastfeeding.

**Results:**

138 pregnant patients with cardiac disease were included: 58 (42%) NHB and 80 (58%) NHW patients. Parity, marital status and insurance were statistically different between groups. NHB patients were more likely to have government insurance compared to NHW patients (77.6% vs. 40%; *p* < 0.001). There was a significant difference in the intent to breastfeed upon admission for the delivery-associated hospitalization (74.2% NHB vs. NHW 91.3%; *p* = 0.01), but not at hospital discharge (84.5% NHB vs. 93.8% NHW; *p* = 0.08). However, breastfeeding rates were significantly lower among NHB patients at the postpartum visit among the entire cohort (38.2% in NHB vs. 61.1% in NHW women; *p* = 0.036) and among those who initiated breastfeeding (35.3% NHB vs. 61.1% NHW, *p* = 0.018).

**Conclusions:**

Despite similar breastfeeding rates at hospital discharge, NHB patients with maternal cardiac disease were less likely to intend to breastfeed at admission and/or continue breastfeeding by the postpartum visits. Qualitative studies understanding these differences are crucial to improve breastfeeding rates, especially for NHB patients with maternal cardiac disease.

**Supplementary Information:**

The online version contains supplementary material available at 10.1007/s40615-024-01933-1.

## Introduction

Breastfeeding has many benefits for both mothers and their babies. The U.S. Department of Health and Human Services reported a decreased risk of childhood asthma, ear infections, gastrointestinal issues, sudden infant death syndrome, respiratory infections, and infant mortality in breastfed babies compared to babies who are formula fed [1–4]. Mothers who breastfeed also experience a myriad of benefits including weight loss and lower rates of type 2 diabetes, breast cancer, endometrial cancer, hypertension, and hyperlipidemia [1–8]. These benefits explain the firmly held recommendation to exclusively breastfeed for 6 months by the American Academy of Pediatrics, the U.S. Department of Health and Human Services, and the Centers for Disease Control (CDC) [1, 9]. However, despite this recommendation, there are documented racial disparities in breastfeeding rates between NHB and NHW women due to multiple factors including the social determinants of health and related factors that contribute to lower rates of breastfeeding [10].

In a study of patients without heart disease, the rate of exclusive breastfeeding after delivery for the first 3 months was 39.1% in NHB women vs. 52.9% in NHW counterparts [10]. Despite benefits of breastfeeding for both mother and baby, U.S. breastfeeding rates among women from all racial/ethnic groups are low compared to the objectives set forth by the U.S. Department of Health and Human Services [2]. NHB women have the lowest rates, and are 2.5 times less likely to breastfeed than NHW women [2]. The decision to breastfeed is based on multiple factors including the availability of resources but also the sociological perception of breastfeeding for each individual [11].

As advancements in medical and surgical treatments have occurred over the years, the number of people with congenital heart disease (CHD) living into adulthood has increased [3, 12, 13]. For instance, survival rate in children born with CHD in the 1950s was only about 15%, which has significantly improved to more than 90% today [14]. The estimated prevalence of adults with CHD is approximately 3000 per million people [15, 16]. As a result, the number of childbearing women with heart disease has increased resulting in more complex pregnancies and an associated increase in morbidity in this patient population [13, 15, 17]. In pregnancy, the cardiovascular system undergoes structural and hemodynamic adaptations to sustain a high-volume load, a change which can increase pregnancy morbidity in all women and especially in those with underlying heart disease.

Therefore, the objective of our study was to examine differences in breastfeeding rates between NHB and NHW women in a cardio-obstetrics program. We hypothesized that there is a difference in breastfeeding rates between NHB and NHW women with heart disease at the time of discharge from the delivery-associated hospitalization.

## Methods

We conducted a retrospective study of a cohort of pregnant patients with maternal cardiac disease managed by the University of Alabama at Birmingham (UAB) Cardio-Obstetrics Program (IRB-300,002,012). The UAB Cardio-Obstetrics Program provides maternal and fetal care services for women with congenital or acquired heart disease. This program is directed by an adult congenital cardiologist and employs a large team including Maternal–Fetal Medicine specialists, cardiologists, pharmacists, obstetric anesthesiologists, and perinatal nurses to provide multidisciplinary care for women with cardiac conditions. Women are cared for in the program after referral by their primary OB provider if they have a diagnosis of cardiac disease prior and/or during pregnancy. Their baseline information and history are collected on first visit to the Cardio-Obstetrics clinic and includes sociodemographic characteristics such as insurance, marital status, and living situation. Of important note, referral to this program may mean complete prenatal care in the program and for some, participation in the program may include co-management of care with the remainder of the care by the OB provider. The choice in clinic was determined by several factors including proximity to the UAB clinic compared to their obstetric provider, planned cardiac support needed. amongst others. Regardless of which one a woman had in her pregnancy; all providers were responsible for discussing routine pregnancy care including mode of feeding after delivery. Women were included in this study if they attended one or more prenatal visits with the UAB Cardio-Obstetrics Program and delivered at the UAB hospital between March 2015 and June 2019. Women were excluded if their ethnicity was any other than NHB or NHW, if their infant feeding modality was not documented, if they chose adoption, or if they delivered at an outside institution. The UAB institutional review board granted approval for this research study.

Of note, The UAB hospital is a designated baby friendly hospital and so all providers and staff receive appropriate training for the maintenance of this designation. Baseline maternal, delivery and neonatal outcomes were abstracted from the medical record by trained health professionals as previously described [18, 19].Additional variables relating to breastfeeding were abstracted by A.R. and A.G.O. Specifically, these included breastfeeding intent at delivery, any breastfeeding at hospital discharge, and any breastfeeding at the postpartum visit. Breastfeeding intent at delivery was defined as self-reported intent to either exclusively or partially breastfeed at admission for the delivery-associated hospitalization. This was abstracted from the history and physical note for the delivery-associated hospitalization. Any breastfeeding at hospital discharge was defined as breastfeeding or expression of breastmilk at discharge from the delivery-associated hospitalization. This variable was abstracted from the mother’s day of discharge progress note from the delivery-associated hospitalization. Breastfeeding at the postpartum visit was defined as a self-report of breastfeeding or expression of breastmilk at the postpartum visit occurring between 4 and 8 weeks postpartum and abstracted from the postpartum visit note.

The primary outcome was the breastfeeding rate of NHB and NHW women with heart disease at the delivery-associated hospital discharge. Secondary outcomes included breastfeeding intent at admission for delivery and breastfeeding rates in women with heart disease at the postpartum visit.

Risk assessment of cardiac disease was determined using the modified World Health Organization (WHO) classification of maternal cardiovascular risk. The modified WHO pregnancy is a tool that risk stratifies cardiovascular disease into 5 groups and informs the health care provider of the frequency of cardiology evaluation recommended. The patients are classified as very low risk (class I), low to moderate risk (class II), high risk (class III) and extremely high risk (class IV), in which pregnancy is contraindicated [20].

### Statistical Analysis

Baseline demographics and study outcomes were compared between NHB and NHW patients using χ² tests of association or Fisher’s exact tests, as appropriate, for categorical variables. Student’s t test and Wilcoxon rank sum tests, as appropriate, were used to evaluate continuous variables. Statistical significance was assessed at a 0.05 level (*p* < 0.05). No adjustments were made for multiple comparisons. All analyses were performed with SAS, version 9.4 (SAS Institute, Cary, NC, USA).

## Results

**Fig. 1:**
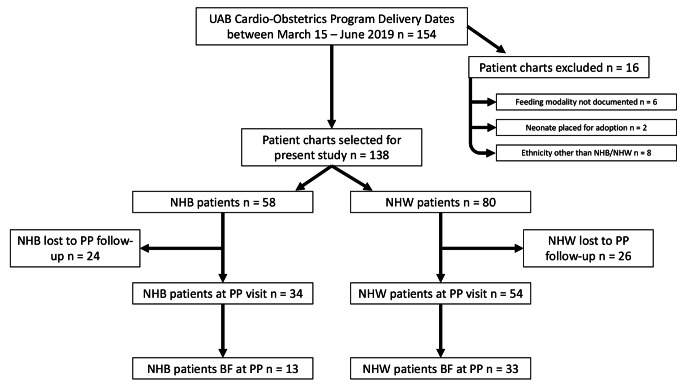
Flowchart showing inclusion and exclusion of participants

Of 150 identified subjects, 58 (38.7%) NHB and 80 (53.3%) NHW patients met inclusion criteria (Fig. 1). Table 1 outlines baseline maternal characteristics for both NHB and NHW mothers with maternal cardiac disease. There was no difference noted in maternal age, gestational age and mode of delivery or comorbidities. However, parity, insurance and marital status were significantly different between groups (Table 1). The number of children (1.4 vs. 0.7; *p* = 0.003) and public insurance (77.6 vs. 40.0%; *p* < 0.001) were higher in NHB vs. NHW patients. While not statistically significant, it should also be noted that NICU admissions were higher among NHB as compared to NHW patients (29.3% vs. 16.3%, *p* = 0.070).

**Table 1:** Baseline characteristics of patients receiving care in a Cardio-Obstetrics Program

CHARACTERISTIC	NHB (*n* = 58)	NHW (*n* = 80)	*p*-value
Maternal age (years)	28.4 ± 6.4	26.5 ± 6.2	0.09
Parity	1.4 ± 1.6	0.7 ± 0.8	< 0.01
BMI (kg/m^2^)	33.4 ± 8.7	31.2 ± 8.4	0.14
Married or living with partner	11 (19.0)	48 (60.8)	< 0.01
Insurance status			< 0.01
Private	8 (13.8)	47 (58.8)	
Public	45 (77.6)	32 (40.0)	
Other	5 (8.6)	1 (1.3)	
GA at delivery	36.4 ± 3.6	37.4 ± 3.2	0.13
Mode of delivery			0.38
Vaginal	29 (50.0)	46 (57.5)	
Cesarean	29 (50.0)	34 (42.5)	
NICU Admission	17 (29.3)	13 (16.3)	0.07
GHTN/Preeclampsia	14 (24.1)	21 (26.3)	0.78
CHTN	12 (20.7)	9 (11.3)	0.13
DM	5 (8.6)	7 (8.8)	0.98
mWHO Class			
I	5 (8.6)	7 (8.8)	0.98
II	11 (19.0)	22 (27.5)	0.25
II– III	10 (17.2)	20 (25.0)	0.28
III	17 (29.3)	19 (23.8)	0.46
IV	15 (25.9)	12 (15.0)	0.11

NHB = Non-Hispanic Black, NHW = Non-Hispanic White, BMI = body mass index, GA = gestational age,

NICU = neonatal intensive care unit, NICU = neonatal intensive care unit GHTN = gestational hypertension, CHTN = chronic hypertension, DM = diabetes mellitus, mWHO = Modified World Health Organization

**Fig. 2:**
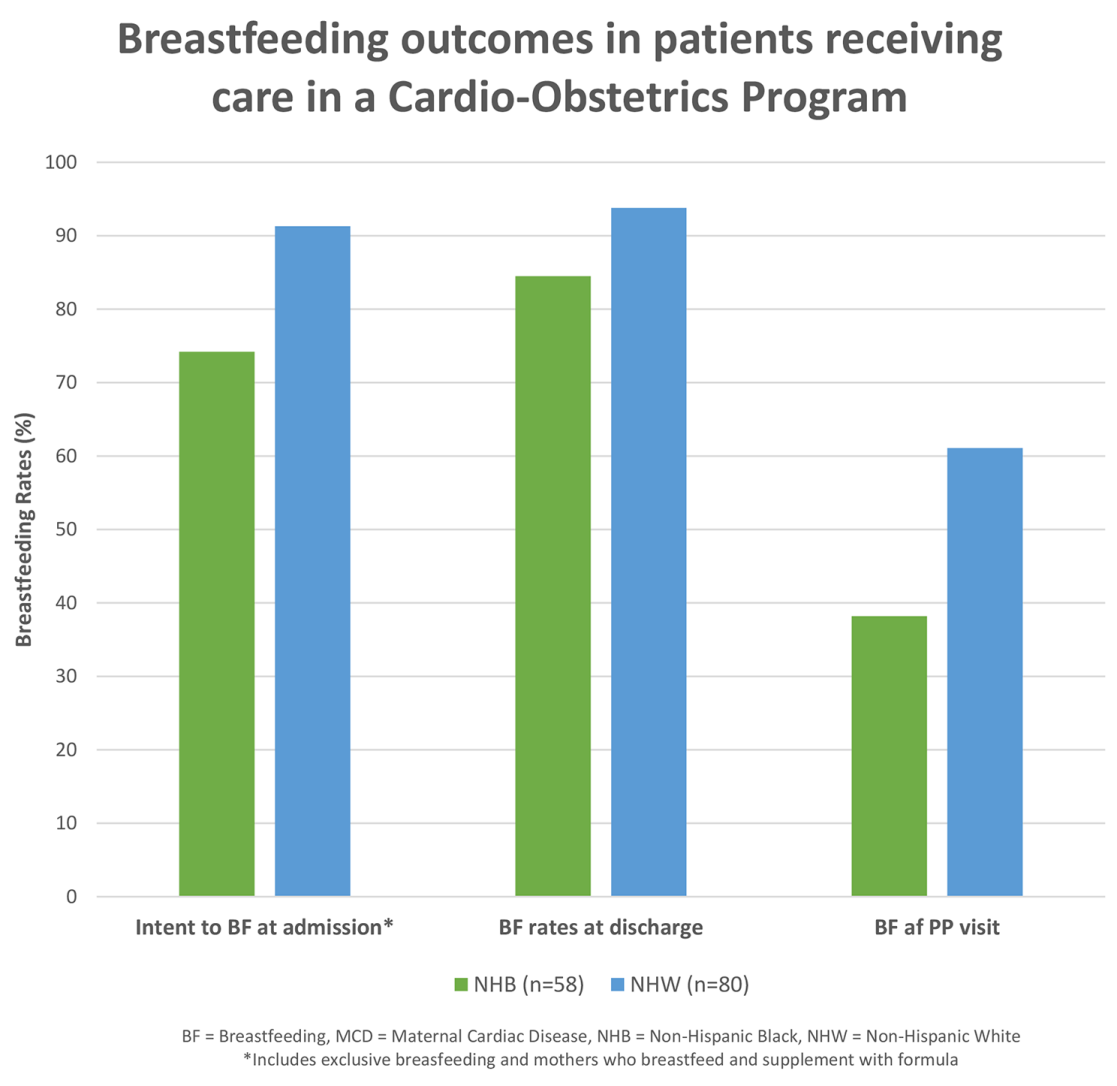
Breastfeeding outcomes at differing time points for patients receiving care in a Cardio Obstetrics program

There was a significant difference between NHB and NHW and their intent to breastfeed at admission for delivery (Table 2). At the time of admission, less than half (46.6%) of NHB women planned to exclusively breastfeed, which was considerably less than the 72.5% of NHW women who intended to do so (*p* = 0.010). When considering any intent to breastfeed on admission for their delivery-associated hospitalization, 74.2% of NHB vs. 91.3% of NHW patients planned to either partially or exclusively breastfeed. This discrepancy was slightly less notable for the rates of breastfeeding at the time of discharge from the delivery-associated hospitalization. When looking at the rate of any breastfeeding at the time of discharge from the delivery-associated hospitalization, 84.5% of the women were NHB compared to 93.8% NHW (*p* = 0.08), although this was not statistically significant. Out of the total cohort of 58 NHB and 80 NHW, only 34 NHB and 54 NHW attended their postpartum visit, as displayed in Fig. 1. Among those who followed up, rates of breastfeeding were NHB 35.3% vs. NHW 61.1% (*p* = 0.010). Breastfeeding rates at differing time points are shown in Fig 2. We compared women who did follow up to those were lost to follow up and found that those attending the postpartum visit had a higher intent to breastfeed at admission for delivery however, this was not statistically significant (*p* = 0.09). We also performed a regression analysis but did not find any variables which were predictive of breastfeeding at discharge (data not shown).

**Table 2:** Breastfeeding outcomes in patients receiving care in a Cardio-Obstetrics Program

OUTCOMES	NHB (*n* = 58)	NHW (*n* = 80)	*p*-value
BF intent at admission for delivery			0.01
Formula feed	8 (13.8)	3 (3.8)	
Both breast and formula	16 (27.6)	15 (18.8)	
Breastfeed	27 (46.6)	58 (72.5)	
Unknown/Undecided	7 (12.1)	4 (5.0)	
BF rates at hospital discharge	49 (84.5)	75 (93.8)	0.08
BF rates at the postpartum visit	13 (38.2)*^†^	33 (61.1)*	0.04
PP BF rates among those who initiate BF	12 (35.3)* ^†^	33 (61.1)*	0.02

BF = Breastfeeding, MCD = Maternal Cardiac Disease, NHB = Non-Hispanic Black,

NHW = Non-Hispanic White, PP = Postpartum

*Women who attended PP visit, NHB *n* = 34, NHW *n* = 54

^†^1 woman elected to begin breastfeeding after hospital discharge

## Discussion

In women with heart disease, significantly more NHW women intended to breastfeed compared to their NHB counterparts at the time of admission for delivery. At the time of discharge from the delivery-associated hospitalization, there was no difference between breastfeeding rates. However, almost twice as many NHW women were still breastfeeding at time of the postpartum visit as compared to NHB women.

These disparities are consistent with populations of women without maternal cardiac disease as fewer women self-identifying as NHB breastfeed [21]. Beauregard et al. found that the differences amongst breastfeeding rates in NHB and NHW women were 14.7% for any breastfeeding and 17% for exclusive breastfeeding at 3 months postpartum. This persisted even at 6 months postpartum with a difference of 17.3% for any breastfeeding and 12.4% for exclusive breastfeeding at 6 months postpartum in NHW and NHB women respectively [21]. Another long-term study by Anstey et al. evaluated breastfeeding initiation and duration amongst infants born between 2010 and 2013. This study also exhibited a divergence between NHB and NHW women with 17.2% fewer NHB women initiating breastfeeding and 8.5% fewer NHB women exclusively breastfeeding at 6 months [22].

There are several theories that may explain this disparity including working conditions, partner support, and insurance coverage. According to the U.S. Bureau of Labor Statistics, NHB women comprise 60% of the workforce, the highest rates among adult women when all races are compared [23]. NHB women are also less likely to be married to or living with their partner. Supporting a single income household as their family’s sole breadwinner makes their return to work critical, consequently shortening their maternity leave [24]. However, this return to work is often met with resource limitations for breastfeeding hindering their continuation in the postpartum period.

Consistent with limited resources, a majority of NHB women in our cohort were supported through public programs such as Medicaid, which despite having preventive services and resources for breastfeeding education, lactation consultations, and supplies to express breastmilk for infants not directly latching, these benefits are not available for most people in states that did not expand Medicaid. The Patient Protection and Affordable Care Act was amended in 2010 to require employers to provide women who were breastfeeding “reasonable break time” to pump and properly store the mother’s milk. However, breastfeeding mothers still experience discrimination in the workplace, receiving negative stigma from coworkers and supervisors that discourages nursing [24]. Additionally, Alabama is one of the states that has not opted for Medicaid expansion at this time. Taken together, cumulatively these factors and discrimination could explain the discrepancy between NHB women who intended to breastfeed at admission, breastfed at delivery-associated hospital discharge, but stopped breastfeeding at their post-partum visit. It should also be noted that while not statistically significant, NHB babies were born approximately one week earlier than NHW babies and had a higher frequency of NICU admissions. Breastfeeding challenges among infants in the NICU have been well described and this could also contribute to some of our findings.

Other studies also reveal breastfeeding racial divergence in the background of chronic disease. Kachoria et al. studied a cohort of women with diabetes and found that breastfeeding initiation rates vary by diabetes status and race. Women with pre-pregnancy diabetes had lower breastfeeding initiation rates and NHB women with pregestational diabetes had the lowest breastfeeding initiation rates overall [25]. A study by Stevens et al. also showed a substantial difference in breastfeeding initiation between NHB and NHW women in a population of women with maternal diabetes, with NHB mothers least likely to breastfeed [26]. Another study by Morrow et al. identified differences between NHB and NHW women with chronic hypertension. At the time of admission for delivery in this population, women reporting NHB status were less likely to breastfeed at the postpartum visit, compared to NHW patients. Their study also followed these women through to 6 months postpartum and noted that this disparity persisted [27].

Our study is unique in that it evaluates an important issue: breastfeeding in the context of health disparities in a unique patient population– patients with the diagnosis of maternal cardiac disease. Additionally, modified WHO (mWHO) classification was assigned by a double board-certified adult congenital cardiologist and were similar between groups, eliminating cardiac disease status as the driver of the disparity. Further, all patients are managed via the same protocols, reducing the chance that care or counseling resulted in disparate findings (Appendix 1).

This study is not without limitations, however. First, we note that our analysis does not include variables that may influence breastfeeding rates such as highest educational level or socioeconomic status at the census tract level which would be important to further understand the subtlety of differences in disparities within each population. Second, we have a relatively small sample size and may not be powered for certain outcomes which are also limited by the inability to control for all variables. Third, our results and conclusions may not be generalizable to other practices or populations as our study did not include any prediction models.

## Conclusion

Racial disparities in breastfeeding practices are a prominent and concerning issue in today’s healthcare system. Our study evaluated racial and health disparities in breastfeeding between NHB and NHW women in the setting of maternal cardiac disease. Our study revealed several key findings, specifically that NHB women with maternal cardiac disease were less likely to intend to breastfeed, and initiate and/or maintain breastfeeding by the postpartum visit. This study identifies the importance of interventions aimed to support women self-reporting minority status so that they and their infants can realize benefits of breastfeeding.

## Appendix 1: Modified WHO (mWHO) Classification)

WHO class I: uncomplicated, small or mild pulmonary stenosis, patent ductus arteriosus and mitral valve prolapse; successfully repaired simple lesions (atrial or ventricular septal defect, patent ductus arteriosus, anomalous pulmonary venous drainage); and atrial or ventricular ectopic beats, isolated.

WHO class II (if otherwise well and uncomplicated): unoperated atrial or ventricular septal defect; repaired tetralogy of Fallot; and most arrhythmias.

WHO class II–III: mild left ventricular impairment; hypertrophic cardiomyopathy; native or tissue valvular heart disease not considered WHO I or IV; Marfan syndrome without aortic dilatation; aorta < 45 mm in aortic disease associated with bicuspid aortic valve; repaired coarctation.

WHO class III: mechanical valve; systemic right ventricle; Fontan circulation; cyanotic heart disease (unrepaired); other complex congenital heart disease; aortic dilatation 40–45 mm in Marfan syndrome; and aortic dilatation 45–50 mm in aortic disease associated with bicuspid aortic valve.

WHO class IV: pulmonary arterial hypertension of any cause; severe systemic ventricular dysfunction (left ventricular ejection fraction < 30%, New York Heart Association III–IV); previous peripartum cardiomyopathy with any residual impairment of left ventricular function; severe mitral stenosis and severe symptomatic aortic stenosis; Marfan syndrome with aorta dilated > 45 mm; aortic dilatation > 50 mm in aortic disease associated with bicuspid aortic valve; and native severe coarctation.

## Electronic Supplementary Material

Below is the link to the electronic supplementary material.

Supplementary Material 1

Supplementary Material 2

Supplementary Material 3
