# Supplementary material for: Racial Disparities in Breastfeeding Rates in Patients with Heart Disease
Source: J Racial Ethn Health Disparities. 2024 Mar 20;12(2):970–6. doi: 10.1007/s40615-024-01933-1 (PMC11913963; doi:10.1007/s40615-024-01933-1)
Supplement: Supplementary file 2 — Supplementary Material 2 [file 40615_2024_1933_MOESM2_ESM.docx]

**Racial disparities in breastfeeding rates in patients with heart disease**

Ayamo Oben, MD^1,2^; Christina T Blanchard, MS^1,3^; Ashton Robinson, BS^1^; Isabel Girling, BS^1^; Joanna M. Joly, MD^1,4^; Marc Cribbs, MD^1,4^; Alan Tita, MD, PhD^1,2,3^; Brian Casey, MD^1,2,3^, Rachel Sinkey MD^1,2,3^

1. University of Alabama at Birmingham Marnix E. Heersink School of Medicine, Birmingham, AL

2. Department of Obstetrics & Gynecology, University of Alabama at Birmingham, Birmingham, AL

3. Center for Women’s Reproductive Health, University of Alabama at Birmingham, Birmingham, AL

4. Department of Medicine, Division of Cardiovascular Disease, University of Alabama at Birmingham, AL

*Corresponding Author: Ayamo Oben MD

The authors report no conflict of interest.

Presented at the Society for Maternal Fetal Medicine 43rd Annual Pregnancy Meeting, February 6 – 11, 2023 as Poster # 676

Corresponding Author: Ayamo G. Oben, The University of Alabama at Birmingham

Address: 1700 6^th^ Ave South, Ste 10270, Birmingham, AL 35233

Phone: 678-313-9795

Fax: 205-975-9858

E-Mail: [aoben2015@gmail.com](mailto:aoben2015@gmail.com)

Rachel Sinkey receives funding from NHLBI K23HL159331

**Abstract**

**Objective**: To evaluate racial disparities in breastfeeding rates in patients with heart disease.

**Study Design:** Retrospective cohort of pregnant patients with maternal cardiac disease managed by a Cardio-Obstetrics program. Patients self-identifying as Non-Hispanic Black (NHB) and Non-Hispanic White (NHW), who attended ≥ 1 prenatal visit at the Cardio-Obstetrics Program and delivered at the same hospital between March 2015 and June 2019 were included. The primary outcome was breastfeeding rate at discharge from the delivery-associated hospitalization. Secondary outcomes included breastfeeding intent on admission and breastfeeding rates at the postpartum visit among patients who initiated breastfeeding.

**Results:** 138 pregnant patients with cardiac disease were included: 58 (42%) NHB and 80 (58%) NHW patients. Parity, marital status and insurance were statistically different between groups. NHB patients were more likely to have government insurance compared to NHW patients (77.6% vs. 40%; p<0.001). There was a significant difference in the intent to breastfeed upon admission for the delivery-associated hospitalization (74.2% NHB vs NHW 91.3%; p=0.01), but not at hospital discharge (84.5% NHB vs. 93.8% NHW; p=0.08). However, breastfeeding rates were significantly lower among NHB patients at the postpartum visit among the entire cohort (38.2% in NHB vs. 61.1% in NHW women; p=0.036) and among those who initiated breastfeeding (35.3 % NHB vs 61.1% NHW, p=0.018).

**Conclusions:** Despite similar breastfeeding rates at hospital discharge, NHB patients with maternal cardiac disease were less likely to intend to breastfeed at admission and/or continue breastfeeding by the postpartum visits. Qualitative studies understanding these differences are crucial to improve breastfeeding rates, especially for NHB patients with maternal cardiac disease.

**Introduction**

Breastfeeding has many benefits for both mothers and their babies. The U.S. Department of Health and Human Services reported a decreased risk of childhood asthma, ear infections, gastrointestinal issues, sudden infant death syndrome, respiratory infections, and infant mortality in breastfed babies compared to babies who are formula fed [1]–[4]. Mothers who breastfeed also experience a myriad of benefits including weight loss and lower rates of type 2 diabetes, breast cancer, endometrial cancer, hypertension, and hyperlipidemia [1]–[8]. These benefits explain the firmly held recommendation to exclusively breastfeed for 6 months by the American Academy of Pediatrics, the U.S. Department of Health and Human Services, and the Centers for Disease Control (CDC) [1], [9]. However, despite this recommendation, there are documented racial disparities in breastfeeding rates between NHB and NHW women due to multiple factors including the social determinants of health and related factors that contribute to lower rates of breastfeeding [10].

In a study of patients without heart disease, the rate of exclusive breastfeeding after delivery for the first 3 months was 39.1% in NHB women vs. 52.9% in NHW counterparts [10]. Despite benefits of breastfeeding for both mother and baby, U.S. breastfeeding rates among women from all racial/ethnic groups are low compared to the objectives set forth by the U.S. Department of Health and Human Services [2]. NHB women have the lowest rates, and are 2.5 times less likely to breastfeed than NHW women [2]. The decision to breastfeed is based on multiple factors including the availability of resources but also the sociological perception of breastfeeding for each individual [11].

As advancements in medical and surgical treatments have occurred over the years, the number of people with congenital heart disease (CHD) living into adulthood has increased [3], [12], [13]. For instance, survival rate in children born with CHD in the 1950s was only about 15%, which has significantly improved to more than 90% today [14]. The estimated prevalence of adults with CHD is approximately 3000 per million people [15], [16]. As a result, the number of childbearing women with heart disease has increased resulting in more complex pregnancies and an associated increase in morbidity in this patient population [13], [15], [17]. In pregnancy, the cardiovascular system undergoes structural and hemodynamic adaptations to sustain a high-volume load, a change which can increase pregnancy morbidity in all women and especially in those with underlying heart disease.

Therefore, the objective of our study was to examine differences in breastfeeding rates between NHB and NHW women in a cardio-obstetrics program. We hypothesized that there is a difference in breastfeeding rates between NHB and NHW women with heart disease at the time of discharge from the delivery-associated hospitalization.

**Methods**

We conducted a retrospective study of a cohort of pregnant patients with maternal cardiac disease managed by the University of Alabama at Birmingham (UAB) Cardio-Obstetrics Program (IRB-300002012). The UAB Cardio-Obstetrics Program provides maternal and fetal care services for women with congenital or acquired heart disease. This program is directed by an adult congenital cardiologist and employs a large team including Maternal–Fetal Medicine specialists, cardiologists, pharmacists, obstetric anesthesiologists, and perinatal nurses to provide multidisciplinary care for women with cardiac conditions. Women are cared for in the program after referral by their primary OB provider if they have a diagnosis of cardiac disease prior and/or during pregnancy. Their baseline information and history are collected on first visit to the Cardio-Obstetrics clinic and includes sociodemographic characteristics such as insurance, marital status, and living situation. Of important note, referral to this program may mean complete prenatal care in the program and for some, participation in the program may include co-management of care with the remainder of the care by the OB provider. The choice in clinic was determined by several factors including proximity to the UAB clinic compared to their obstetric provider, planned cardiac support needed. amongst others. Regardless of which one a woman had in her pregnancy; all providers were responsible for discussing routine pregnancy care including mode of feeding after delivery. Women were included in this study if they attended one or more prenatal visits with the UAB Cardio-Obstetrics Program and delivered at the UAB hospital between March 2015 and June 2019. Women were excluded if their ethnicity was any other than NHB or NHW, if their infant feeding modality was not documented, if they chose adoption, or if they delivered at an outside institution. The UAB institutional review board granted approval for this research study.

The UAB hospital is a designated baby friendly hospital and so all providers and staff receive appropriate training for the maintenance of this designation, Baseline maternal, delivery and neonatal outcomes were abstracted from the medical record by trained health professionals as previously described [18], [19].Additional variables relating to breastfeeding were abstracted by A.R. and A.G.O. Specifically, these included breastfeeding intent at delivery, any breastfeeding at hospital discharge, and any breastfeeding at the postpartum visit. Breastfeeding intent at delivery was defined as self-reported intent to either exclusively or partially breastfeed at admission for the delivery-associated hospitalization. This was abstracted from the history and physical note for the delivery-associated hospitalization. Any breastfeeding at hospital discharge was defined as breastfeeding or expression of breastmilk at discharge from the delivery-associated hospitalization. This variable was abstracted from the mother’s day of discharge progress note from the delivery-associated hospitalization. Breastfeeding at the postpartum visit was defined as a self-report of breastfeeding or expression of breastmilk at the postpartum visit occurring between 4 – 8 weeks postpartum and abstracted from the postpartum visit note.

The primary outcome was the breastfeeding rate of NHB and NHW women with heart disease at the delivery-associated hospital discharge. Secondary outcomes included breastfeeding intent at admission for delivery and breastfeeding rates in women with heart disease at the postpartum visit.

Risk assessment of cardiac disease was determined using the modified World Health Organization (WHO) classification of maternal cardiovascular risk. The modified WHO pregnancy is a tool that risk stratifies cardiovascular disease into 5 groups and informs the health care provider of the frequency of cardiology evaluation recommended. The patients are classified as very low risk (class I), low to moderate risk (class II), high risk (class III) and extremely high risk (class IV), in which pregnancy is contraindicated [20].

**Statistical analysis**

Baseline demographics and study outcomes were compared between NHB and NHW patients using χ² tests of association or Fisher’s exact tests, as appropriate, for categorical variables. Student’s t test and Wilcoxon rank sum tests, as appropriate, were used to evaluate continuous variables. Statistical significance was assessed at a 0.05 level (p<0.05). No adjustments were made for multiple comparisons. All analyses were performed with SAS, version 9.4 (SAS Institute, Cary, NC, USA).

**Results**

Of 150 identified subjects, 58 (38.7%) NHB and 80 (53.3%) NHW patients met inclusion criteria (Figure 1). Table 1 outlines baseline maternal characteristics for both NHB and NHW mothers with maternal cardiac disease. There was no difference noted in maternal age, gestational age and mode of delivery or comorbidities. However, parity, insurance and marital status were significantly different between groups (Table 1). The number of children (1.4 vs. 0.7; p=0.003) and public insurance (77.6 vs. 40.0%; p<0.001) were higher in NHB vs NHW patients. While not statistically significant, it should also be noted that NICU admissions were higher among NHB as compared to NHW patients (29.3% vs. 16.3%, p = 0.070).

There was a significant difference between NHB and NHW and their intent to breastfeed at admission for delivery (Table 2). At the time of admission, less than half (46.6%) of NHB women planned to exclusively breastfeed, which was considerably less than the 72.5% of NHW women who intended to do so (p=0.010). When considering any intent to breastfeed on admission for their delivery-associated hospitalization, 74.2% of NHB vs. 91.3% of NHW patients planned to either partially or exclusively breastfeed. This discrepancy was slightly less notable for the rates of breastfeeding at the time of discharge from the delivery-associated hospitalization. When looking at the rate of any breastfeeding at the time of discharge from the delivery-associated hospitalization, 84.5% of the women were NHB compared to 93.8% NHW (p=0.08), although this was not statistically significant. Out of the total cohort of 58 NHB and 80 NHW, only 34 NHB and 54 NHW attended their postpartum visit, as displayed in Figure 1. Among those who followed up, rates of breastfeeding were NHB 35.3% vs. NHW 61.1% (p=0.010). We compared women who did follow up to those were lost to follow up and found that those attending the postpartum visit had a higher intent to breastfeed at admission for delivery however, this was not statistically significant (p=0.09). We also performed a regression analysis but did not find any variables which were predictive of breastfeeding at discharge (data not shown).

**Discussion**

In women with heart disease, significantly more NHW women intended to breastfeed compared to their NHB counterparts at the time of admission for delivery. At the time of discharge from the delivery-associated hospitalization, there was no difference between breastfeeding rates. However, almost twice as many NHW women were still breastfeeding at time of the postpartum visit as compared to NHB women.

These disparities are consistent with populations of women without maternal cardiac disease as fewer women self-identifying as NHB breastfeed [21]. Beauregard et al. found that the differences amongst breastfeeding rates in NHB and NHW women were 14.7% for any breastfeeding and 17% for exclusive breastfeeding at 3 months postpartum. This persisted even at 6 months postpartum with a difference of 17.3% for any breastfeeding and 12.4% for exclusive breastfeeding at 6 months postpartum in NHW and NHB women respectively [21]. Another long-term study by Anstey et al. evaluated breastfeeding initiation and duration amongst infants born between 2010 – 2013. This study also exhibited a divergence between NHB and NHW women with 17.2% fewer NHB women initiating breastfeeding and 8.5% fewer NHB women exclusively breastfeeding at 6 months [22].

There are several theories that may explain this disparity including working conditions, partner support, and insurance coverage. According to the U.S. Bureau of Labor Statistics, NHB women comprise 60% of the workforce, the highest rates among adult women when all races are compared [23]. NHB women are also less likely to be married to or living with their partner. Supporting a single income household as their family’s sole breadwinner makes their return to work critical, consequently shortening their maternity leave [24]. However, this return to work is often met with resource limitations for breastfeeding hindering their continuation in the postpartum period.

Consistent with limited resources, a majority of NHB women in our cohort were supported through public programs such as Medicaid, which despite having preventive services and resources for breastfeeding education, lactation consultations, and supplies to express breastmilk for infants not directly latching, these benefits are not available for most people in states that did not expand Medicaid. The Patient Protection and Affordable Care Act was amended in 2010 to require employers to provide women who were breastfeeding “reasonable break time” to pump and properly store the mother’s milk. However, breastfeeding mothers still experience discrimination in the workplace, receiving negative stigma from coworkers and supervisors that discourages nursing [24]. Additionally, Alabama is one of the states that has not opted for Medicaid expansion at this time. Taken together, cumulatively these factors and discrimination could explain the discrepancy between NHB women who intended to breastfeed at admission, breastfed at delivery-associated hospital discharge, but stopped breastfeeding at their post-partum visit. It should also be noted that while not statistically significant, NHB babies were born approximately one week earlier than NHW babies and had a higher frequency of NICU admissions. Breastfeeding challenges among infants in the NICU have been well described and this could also contribute to some of our findings.

Other studies also reveal breastfeeding racial divergence in the background of chronic disease. Kachoria et al. studied a cohort of women with diabetes and found that breastfeeding initiation rates vary by diabetes status and race. Women with pre-pregnancy diabetes had lower breastfeeding initiation rates and NHB women with pregestational diabetes had the lowest breastfeeding initiation rates overall [25]. A study by Stevens et al. also showed a substantial difference in breastfeeding initiation between NHB and NHW women in a population of women with maternal diabetes, with NHB mothers least likely to breastfeed [25]. Another study by Morrow et al. identified differences between NHB and NHW women with chronic hypertension. At the time of admission for delivery in this population, women reporting NHB status were less likely to breastfeed at the postpartum visit, compared to NHW patients. Their study also followed these women through to 6 months postpartum and noted that this disparity persisted [27].

Our study is unique in that it evaluates an important issue, breastfeeding in the context of health disparities in a unique patient population – patients with the diagnosis of maternal cardiac disease. Additionally, modified WHO (mWHO) classification was assigned by a double board-certified adult congenital cardiologist and were similar between groups, eliminating cardiac disease status as the driver of the disparity. Further, all patients are managed via the same protocols, reducing the chance that care or counseling resulted in disparate findings (Appendix 1).

This study is not without limitations, however. First, we note that our analysis does not include variables that may influence breastfeeding rates such as highest educational level or socioeconomic status at the census tract level which would be important to further understand the subtlety of differences in disparities within each population. Second, we have a relatively small sample size and may not be powered for certain outcomes which are also limited by the inability to control for all variables. Third, our results and conclusions may not be generalizable to other practices or populations as our study did not include any prediction models.

**Conclusion**

Racial disparities in breastfeeding practices are a prominent and concerning issue in today’s healthcare system. Our study evaluated racial and health disparities in breastfeeding between NHB and NHW women in the setting of maternal cardiac disease. Our study revealed several key findings, specifically that NHB women with maternal cardiac disease were less likely to intend to breastfeed, and initiate and/or maintain breastfeeding by the postpartum visit. This study identifies the importance of interventions aimed to support women self-reporting minority status so that they and their infants can realize benefits of breastfeeding.

**References:**

[1] K. Kirksey, “A social history of racial disparities in breastfeeding in the United States,” Soc Sci Med, vol. 289, Nov. 2021, doi: 10.1016/j.socscimed.2021.114365.

[2] K. M. Jones, M. L. Power, J. T. Queenan, and J. Schulkin, “Racial and ethnic disparities in breastfeeding,” Breastfeeding Medicine, vol. 10, no. 4. Mary Ann Liebert Inc., pp. 186–196, May 01, 2015. doi: 10.1089/bfm.2014.0152.

[3] Y. Matsuzaka et al., “Breastfeeding and postpartum outcomes among women with congenital heart disease,” International Journal of Cardiology Congenital Heart Disease, vol. 4, p. 100167, Aug. 2021, doi: 10.1016/j.ijcchd.2021.100167.

[4] K. v Chiang, R. Li, E. H. Anstey, and C. G. Perrine, “Morbidity and Mortality Weekly Report Racial and Ethnic Disparities in Breastfeeding Initiation-United States, 2019,” 2021. [Online]. Available: https://www.cdc.gov/mmwr/mmwr_continuingEducation.html

[5] A. M. Stuebe et al., “Duration of lactation and incidence of maternal hypertension: A longitudinal cohort study,” Am J Epidemiol, vol. 174, no. 10, pp. 1147–1158, Nov. 2011, doi: 10.1093/aje/kwr227.

[6] E. B. Schwarz et al., “Duration of lactation and risk factors for maternal cardiovascular disease,” Obstetrics and Gynecology, vol. 113, no. 5, pp. 974–982, May 2009, doi: 10.1097/01.AOG.0000346884.67796.ca.

[7] I. E. Hatsu, D. M. McDougald, and A. K. Anderson, “Effect of infant feeding on maternal body composition,” Int Breastfeed J, vol. 3, Aug. 2008, doi: 10.1186/1746-4358-3-18.

[8] M. P. Jarlenski, W. L. Bennett, S. N. Bleich, C. L. Barry, and E. A. Stuart, “Effects of breastfeeding on postpartum weight loss among U.S. women,” Prev Med (Baltim), vol. 69, pp. 146–150, Dec. 2014, doi: 10.1016/j.ypmed.2014.09.018.

[9] A. I. Eidelman and R. J. Schanler, “Breastfeeding and the use of human milk,” Pediatrics, vol. 129, no. 3. Mar. 2012. doi: 10.1542/peds.2011-3552.

[10] A. F. Louis-Jacques et al., “Historical Antecedents of Breastfeeding for African American Women: from the Pre-Colonial Period to the Mid-Twentieth Century,” Journal of Racial and Ethnic Health Disparities, vol. 7, no. 5. Springer, pp. 1003–1012, Oct. 01, 2020. doi: 10.1007/s40615-020-00727-5.

[11] Carter, S. K., & Anthony, A. K. (2015). Good, Bad, and Extraordinary Mothers: Infant Feeding and Mothering in African American Mothers’ Breastfeeding Narratives. Sociology of Race and Ethnicity, 1(4), 517-531. https://doi.org/10.1177/2332649215581664

[12] K. Niwa, “Adult congenital heart disease with pregnancy,” Korean Circulation Journal, vol. 48, no. 4. Korean Society of Circulation, pp. 251–276, Apr. 01, 2018. doi: 10.4070/kcj.2018.0070.

[13] K. Kearney, D. Zentner, and R. Cordina, “Management of Maternal Complex Congenital Heart Disease During Pregnancy,” Current Heart Failure Reports, vol. 18, no. 6. Springer, pp. 353–361, Dec. 01, 2021. doi: 10.1007/s11897-021-00534-x.

[14] M. Brida and M. A. Gatzoulis, “Adult congenital heart disease: Past, present and future,” Acta Paediatr, vol. 108, no. 10, pp. 1757–1764, 2019, doi: https://doi.org/10.1111/apa.14921.

[15] E. Yucel and D. DeFaria Yeh, “Pregnancy in Women with Congenital Heart Disease,” Current Treatment Options in Cardiovascular Medicine, vol. 19, no. 9. Springer Healthcare, Sep. 01, 2017. doi: 10.1007/s11936-017-0572-0.

[16] T. van der Bom, B. J. Bouma, F. J. Meijboom, A. H. Zwinderman, and B. J. M. Mulder, “The prevalence of adult congenital heart disease, results from a systematic review and evidence based calculation,” Am Heart J, vol. 164, no. 4, pp. 568–575, Oct. 2012, doi: 10.1016/j.ahj.2012.07.023.

[17] C. A. Warnes, “Pregnancy and delivery in women with congenital heart disease,” Circulation Journal, vol. 79, no. 7. Japanese Circulation Society, pp. 1416–1421, Jun. 09, 2015. doi: 10.1253/circj.CJ-15-0572.

[18] R. G. Sinkey et al., “The effects of offering immediate postpartum placement of IUDs and implants to pregnant patients with heart disease,” Contraception, vol. 105, pp. 55–60, Jan. 2022, doi: 10.1016/j.contraception.2021.09.005.

[19] A. G. Oben et al., “Racial disparities in reliable contraceptive use in women with heart disease*,” European Journal of Contraception and Reproductive Health Care, vol. 27, no. 3, pp. 174–179, 2022, doi: 10.1080/13625187.2021.2010042.

[20] ACOG Practice Bulletin No. 212 Summary: Pregnancy and Heart Disease. Obstetrics & Gynecology 133(5):p 1067-1072, May 2019. | DOI: 10.1097/AOG.0000000000003244

21] J. L. Beauregard, H. C. Hamner, J. Chen, W. Avila-Rodriguez, L. D. Elam-Evans, and C. G. Perrine, “Morbidity and Mortality Weekly Report Racial Disparities in Breastfeeding Initiation and Duration Among U.S. Infants Born in 2015.” [Online]. Available: https://www.healthypeople.gov/2020/topics-objectives/topic/maternal-infant-

[22] E. H. Anstey, J. Chen, L. D. Elam-Evans, and C. G. Perrine, “Racial and Geographic Differences in Breastfeeding — United States, 2011–2015,” 2017. [Online]. Available: http://empowerbreastfeeding.org.

[23] U.S. Bureau of Labor Statistics, “Labor force statistics by race and ethnicity, 2020,” Bureau of Labor Statistics, 2020. https://www.bls.gov/opub/reports/race-and-ethnicity/2020/#:~:text=Among%20adult%20women%2C%20Blacks%20(60.7,and%20Whites%20(56.8%20percent). (accessed Jun. 01, 2023).

[24] P. L. F. Rippeyoung and M. C. Noonan, “Is breastfeeding truly cost free? Income consequences of breastfeeding for women,” Am Sociol Rev, vol. 77, no. 2, pp. 244–267, 2012, doi: 10.1177/0003122411435477.

[25] R. Kachoria and R. Oza-Frank, “Differences in Breastfeeding Initiation by Maternal Diabetes Status and Race, Ohio 2006–2011,” Matern Child Health J, vol. 18, no. 9, pp. 2226–2235, Oct. 2014, doi: 10.1007/s10995-014-1472-5.

[26] D. R. Stevens et al., “Breastfeeding initiation as related to the interaction of race/ethnicity and maternal diabetes,” Breastfeeding Medicine, vol. 14, no. 9, pp. 630–639, Nov. 2019, doi: 10.1089/bfm.2019.0065.

[27] A. L. Morrow et al., “Breastfeeding Disparities and Their Mediators in an Urban Birth Cohort of Black and White Mothers,” Breastfeeding Medicine, vol. 16, no. 6, pp. 452–462, Jun. 2021, doi: 10.1089/bfm.2020.0327.

**Declarations**

**Funding**

Ayamo Oben declares no financial interests. Rachel Sinkey receives funding from NHLBI K23HL159331

**Competing Interests**

The authors have no relevant financial or non-financial interests to disclose.

**Author Contributions**

All authors contributed to the study conception and design. Material preparation, data collection and analysis were performed by Ayamo Oben, Ashton Robinson and Rachel Sinkey. The first draft of the manuscript was written by Ayamo Oben and all authors commented on previous versions of the manuscript. All authors read and approved the final manuscript.

**Ethics approval**

This study was performed in line with the principles of the Declaration of Helsinki. Approval was granted by the Internal Board Review of University of Alabama.

**Consent to participate**

Informed consent was obtained from all individual participants included in the study.

**Consent to publish**

The authors affirm that human research participants provided informed consent for publication.

Appendix 1: Modified WHO (mWHO) classification)

WHO class I: uncomplicated, small or mild pulmonary stenosis, patent ductus arteriosus and mitral valve prolapse; successfully repaired simple lesions (atrial or ventricular septal defect, patent ductus arteriosus, anomalous pulmonary venous drainage); and atrial or ventricular ectopic beats, isolated.

WHO class II (if otherwise well and uncomplicated): unoperated atrial or ventricular septal defect; repaired tetralogy of Fallot; and most arrhythmias.

WHO class II–III: mild left ventricular impairment; hypertrophic cardiomyopathy; native or tissue valvular heart disease not considered WHO I or IV; Marfan syndrome without aortic dilatation; aorta <45 mm in aortic disease associated with bicuspid aortic valve; repaired coarctation.

WHO class III: mechanical valve; systemic right ventricle; Fontan circulation; cyanotic heart disease (unrepaired); other complex congenital heart disease; aortic dilatation 40–45 mm in Marfan syndrome; and aortic dilatation 45–50 mm in aortic disease associated with bicuspid aortic valve.

WHO class IV: pulmonary arterial hypertension of any cause; severe systemic ventricular dysfunction (left ventricular ejection fraction <30%, New York Heart Association III–IV); previous peripartum cardiomyopathy with any residual impairment of left ventricular function; severe mitral stenosis and severe symptomatic aortic stenosis; Marfan syndrome with aorta dilated >45 mm; aortic dilatation >50 mm in aortic disease associated with bicuspid aortic valve; and native severe coarctation.
