# Supplementary material for: Racial Disparities in Breastfeeding Rates in Patients with Heart Disease
Source: J Racial Ethn Health Disparities. 2024 Mar 20;12(2):970–6. doi: 10.1007/s40615-024-01933-1 (PMC11913963; doi:10.1007/s40615-024-01933-1)
Supplement: Supplementary file 3 — Supplementary Material 3 [file 40615_2024_1933_MOESM3_ESM.docx]

Journal of Racial and Ethnic Health Disparities

Dear Editor in Chief Laurencin,

Thank you very much for the opportunity to submit our revised manuscript "**Racial disparities in breastfeeding rates in patients with heart disease**" (reference number REHD-D-23-00823) for publication consideration in your esteemed Journal of Racial and Ethnic Health Disparities.

We greatly appreciate your comments and those of the reviewers that have strengthened the quality of the manuscript.

Thank you very much for this opportunity; we hope the quality of the manuscript will be found suitable for publication after modifications and changes made.

Sincerely,

Ayamo Oben, MD

Reviewer #1: The manuscript evaluated racial disparities in breastfeeding rates in patients with heart disease. This topic is very important and a great fit for the journal, however there are some minor issues that need to be addressed. There are some points that need clarity and unpacking to provide the reader more context. Overall, the manuscript was interesting and a good read.

Thank you for your salient perspective. We have made the recommended changes to produce an even better read.


Reviewer #3: Thank you for the opportunity to review your paper. This well-written paper adds to the growing literature surrounding racial disparities in breastfeeding. The study's primary aim was to examine the differences in breastfeeding rates between non-Hispanic black and non-Hispanic white women with heart disease who participated in a cardio-obstetrics program prior to delivery.
However, I have a few comments that I believe should be considered.
Introduction
I think it would help the reader if there were some more information in the Introduction. While included in the Discussion, I think potential reasons for disparities in breastfeeding between White and Black women should be included here.
Good, bad, and extraordinary mothers: Infant feeding and mothering in African American mothers' breastfeeding narratives (Carter et al., 2015)
Racial and ethnic disparities in U.S. breastfeeding and implications for maternal and child health outcomes (Louis-Jacques et al., 2017)
How are pregnancies among women with CHD more complex? What increased morbidity? Has previous literature investigated if this has effected breastfeeding rates? What are the breastfeeding recommendations for women with CHD?
I also think it would be interesting to know why the hypothesis is that there is no difference in breastfeeding rates, especially since in the general (non-CHD) population there are.

Thank you for these important highlights. We have made these changes and added more information into our introduction. As it relates to our hypothesis, we have also modified this sentence to reflect the current trends in breastfeeding.

Methods
If the IRB approval has a number, I think it should be included.
What are the care services provided by the Cardio-Obstetrics Program during the prenatal visits? How many prenatal visits are standard? When are women included in the program? Are they referred by their OB? What sociodemographic variables were extracted, and how?
What was the average number of cardio-obstetrics program prenatal visits? Did you control for this during the analyses? If not, I think this should be mentioned in the Limitations.

Thank you for this recommendation. We have included the IRB approval number in our manuscript. We have also included additional information about the clinic including how a woman gets included in the program.

Additionally, we have provided an additional limitation to our analysis as we did not control for the number of prenatal visits to this program.

Discussion
p. 11, line 24: What does mWHO stand for?
Does the literature on breastfeeding rates explain why women with chronic diseases may have lower breastfeeding initiation rates?
Thank you for your insight and this question. mWHO stands for modified WHO classification. We have included this description in the narrative. There is literature that does discuss that women with chronic illnesses initiate some form of breastfeeding similar to their peers but they have been found to have a higher cessation rate (prior to 6 months). However, these studies have highlighted the need for additional investigation to determine the reasons for this disparity.


Reviewer #4: This manuscript describes a niche population of patients with heart disease who breastfeed. While this study offers a unique lens, I have a number of concerns regarding the interpretation and value of findings. I've outlined these and other areas for revision below:

Abstract
1. Page 2, Line 46: add "to breastfeeding at admission" after "intend" to make this statement clear.

Thank you for this suggestion for additional clarity. This change has been made.

Introduction
2. Page 3, Line 33: remove the first mention of "in NHB women" as this is stated twice.
3. Page 4, Lines 12-17: given the authors' review of past literature and trends, both here and in the Discussion section (pages 9-10, Lines 45-end of paragraph), it is not clear why their hypothesis would be that there would be no difference in breastfeeding rates by race. The authors must justify why their hypothesis goes against the findings from prior studies and breastfeeding trends in the general population.

Thank you for these important highlights. We have made these changes to our introduction. As it relates to our hypothesis, we have also modified this hypothesis to be more reflective of the current trends in breastfeeding.

Methods
4. Page 5, Lines 6-33: more information is needed about the hospital from which the sample is drawn. For example, is this a Baby Friendly Hospital? What kind of breastfeeding training do the healthcare providers have? What are the breastfeeding and related protocols? What is the general rate of breastfeeding at this hospital?

Thank you for this suggestion. We have included more information about the hospital specifically its information surrounding breastfeeding practices.

5. Page 6, Line 35: for statistical significance, was this assessed as p< 0.05 or p=/< 0.05? It is not clear from the statement made.
6. All variables listed in the Tables should be explained in the Methods section. For example, what is the mWHO class?

Thank you for this important request for clarification. We have included the significance of the p-value as less than 0.05 and included this in the methods section. We have also included a description of mWHO classes in the narrative. Briefly, mWHO class stands for modified WHO classification and is a risk stratification tool used in women with cardiac disease in pregnancy.

Results
7. Page 7, Lines 11-14: findings regarding tobacco and alcohol use are reported in the narrative but are not listed in Table 1. These data must be added to the table.

We apologize for this failure to include. After review of our data, we realize that this was an overstatement as our data on tobacco and alcohol was not complete and so not included in our Table 1.

8. Page 7, Lines 19-21, Lines 26-33: the authors incorrectly state the interpretation of a chi square test. Given that insurance status and intent to breastfeed are variables with more than two categories, the only interpretation that can be made is that the distribution of categories for these variables is significantly different between NHB and NHW. It cannot be said that one category within the variable (e.g., public insurance) is significantly different for one group vs. the other.
9. Page 7, Line 26: extra ' with the word "women's'"

Thank you for this feedback. This change has been made.

10. Page 7, Line 28: the callout should be for Table 2

Thank you for this relevant recommendation. This change has been made.

11. Page 7: throughout this section, there is discrepancy in how percentages are reported (either whole number or to the nearest tenth). Additionally, p-values are sometimes shown to the nearest hundredth, while others to the nearest thousandth. The authors should be consistent in their reporting throughout, and make sure rounding is accurate. See, for example, Page 7 Line 46 (should be 85%).

Thank you for this recommendation. We have made all appropriate changes to ensure consistency in decimal places with percentages to the nearest tenth and p values to the nearest thousandth.

12. The authors note in Figure 1 that 50 people were lost to follow-up. Have those 50 people been compared to the 88 patients who had a post-partum visit in terms of demographic variables and dependent variables (i.e., intent to breastfeed and breastfeeding at discharge)? Are there any patterns? Potential for bias? It is difficult to interpret the post-partum visit difference otherwise. For example, what % of those lost-to-followup initiative or intended to breastfeed, and was that statistically different than those who stayed in the study?

Thank you for highlighting this excellent point as it relates to the interpretation of the data for the postpartum, visit. We performed an analysis as can be seen below and found that those attending postpartum visit had lower parity, higher frequencies of being married, having private insurance, II-III mWHO Class, and higher intent to breastfeed at admission for delivery.

However, despite this, the difference between these groups as it relates to intent to breastfeed was not statistically significant (p: 0.09).

We have included a statement of this in our manuscript.

| Table 1. Baseline characteristics of patients receiving care in a Cardio-Obstetrics Program | | | |
| --- | --- | --- | --- |
| CHARACTERISTIC | PP Visit (n=88) | No PP Visit (n=50) | p-value |
| Maternal age (years) | 27.6 ± 6.1 | 26.7 ± 6.8 | 0.41 |
| Parity | 0.8 ± 0.9 | 1.4 ± 1.7 | 0.012 |
| BMI (kg/m^2^) | 32.4 ± 8.5 | 31.6 ± 8.8 | 0.63 |
| Married or living with partner | 43 (49.4) | 16 (32.0) | 0.047 |
| Insurance status |  |  | 0.006 |
| Private | 42 (47.7) | 13 (26.0) |  |
| Public | 45 (51.1) | 32 (64.0) |  |
| Other | 1 (1.1) | 5 (10.0) |  |
| GA at delivery | 37.1 ± 3.0 | 36.7 ± 4.0 | 0.54 |
| Mode of delivery |  |  | 0.95 |
| Vaginal | 48 (54.6) | 27 (54.0) |  |
| Cesarean | 40 (45.5) | 23 (46.0) |  |
| NICU Admission | 19 (21.6) | 11 (22.0) | 0.96 |
| GHTN/Preeclampsia | 26 (29.6) | 9 (18.0) | 0.13 |
| CHTN | 14 (15.9) | 7 (14.0) | 0.76 |
| DM | 7 (8.0) | 5 (10.0) | 0.76 |
| mWHO Class |  |  |  |
| I | 11 (12.5) | 1 (2.0) | 0.06 |
| II | 21 (23.9) | 12 (24.0) | 0.99 |
| II – III | 14 (15.9) | 16 (32.0) | 0.028 |
| III | 26 (29.6) | 10 (20.0) | 0.22 |
| IV | 16 (18.2) | 11 (22.0) | 0.59 |
| BF intent at admission for delivery |  |  | 0.009 |
| Formula feed | 4 (4.6) | 7 (14.0) |  |
| Both breast and formula | 22 (25.0) | 9 (18.0) |  |
| Breastfeed | 59 (67.1) | 26 (52.0) |  |
| Unknown/Undecided | 3 (3.4) | 8 (16.0) |  |
| BF rates at hospital discharge | 82 (93.2) | 42 (84.0) | 0.09 |
| NHB = Non-Hispanic Black, NHW = Non-Hispanic White, BMI = body mass index, GA = gestational age,  NICU = neonatal intensive care unit, NICU = neonatal intensive care unit GHTN = gestational hypertension, CHTN = chronic hypertension, DM = diabetes mellitus, mWHO = Modified World Health Organization | | | |

13. The authors only report on chi square tests of independence between dependent variables, so while patterns can be seen, there is little value to the findings. In other words, it is impossible to know if race matters more than other factors like parity, marital status, insurance status, or intent to breastfeed upon admission.  Can the authors perform a regression analysis to see which variables, if any, are predictive of breastfeeding at discharge?

Thank you for this excellent suggestion. We performed a regression analysis but did not find any variables which were predictive of breastfeeding at discharge. Of important note, our study focuses on the disparities between the groups of a specific population. Future studies are planned in which we intend to look at variables that are predictive of breastfeeding in this population.

| Table 3: Unadjusted and adjusted odd ratio and confidence intervals for prevalence of self-reported breastfeeding at discharge (4-6 weeks from birth) (Breastfeeding at discharge – yes/no) | | | | | | | |
| --- | --- | --- | --- | --- | --- | --- | --- |
| Variable | Unadjusted OR | | | | Adjusted OR | |  |
|  | OR | CI | P-value | OR | | CI | P-value |
| Race |  |  |  |  | |  |  |
| NHB | 0.36 | 0.12-1.15 | 0.08 | 1.47 | | 0.19-11.65 | 0.40 |
| NHW (Ref) |  |  |  |  | |  |  |
| Parity | 0.79 | 0.55-1.13 | 0.19 | 0.84 | | 0.45-1.56 | 0.49 |
| Married or living with partner (Ref) | 2.02 | 0.60-6.80 | 0.26 | 1.21 | | 0.15-9.60 | 0.89 |
| Insurance status |  |  |  |  | |  |  |
| Public (Ref) vs Private/Other | 3.22 | 0.86-12.11 | 0.08 | 1.40 | | 0.17-11.49 | 0.27 |
| ~~Intent to breastfeed on admission~~ |  |  |  |  | |  |  |
| ~~Any breastfeeding vs Formula (Ref)~~ | ~~126.00~~ | ~~20.25-784.03~~ | ~~<0.001~~ | ~~119.00~~ | | ~~16.88-838.80~~ | ~~<0.001~~ |

BF intent and BF at discharge are correlated with each other, (0.7272, p<0.001) and so these were removed from the model

Discussion
14. Page 8, Line 14: "However, twice as many NHW women..." is not an accurate statement. 61% is not twice 35%.

Thank you for this highlight. We have modified the wording in this sentence.

15. Page 8, Lines 43-44: can the authors use their data to see if these or other factors explain the difference in breastfeeding rates?
16. Page 8, Line 51: missing the word "are" before compared.

Thank you for your salient perspective. We have made the recommended changes to produce a better read.

17: Page 9, Lines 36-38: NHB is listed twice, though one should be NHW.

Thank you for this relevant point. We have made this change.

18. An interesting finding that was not discussed was the increase in rates between intent to breastfeed at admission and any breastfeeding at discharge. The authors should acknowledge this and discuss the possible/likely reason(s) behind this increase.
19. Page 10, Line 24: mWHO classification is discussed here but never in the Methods or Results. New findings should not be presented in the Discussion.
20. Page 10, Line 34-47: the biggest limitation to this study, beyond what is already listed, is that the results are purely descriptive. No inferential statistics were used to determine which, if any, variables are most predictive of the dependent variables.

Thank you for this highlight. This has been included as a limitation. While we did not include any prediction models, descriptive statistics still provide value to the literature especially in such a specific population.

Table 2
21. According to this table, there were 13 women who breastfed at postpartum, but only 12 initiated breastfeeding at discharge. Does this mean that 1 person was discharged with no breastfeeding, but then began breastfeeding after hospital discharge? Please clarify with a footnote in the table.

Thank you for your salient perspective. We have made the recommended changes for clarification.


22. It should be mentioned somewhere, whether here or in the manuscript narrative, that a greater proportion of NHW patients came for the postpartum visit compared to NHB.

Thank you for this suggestion. We have included this in our manuscript.

23. Something is confusing about the % calculated for post-partum breastfeeding rates among those who initiate breastfeeding. Given this description, the denominator should be the # of people who initiated breastfeeding out of all those who came for their post-partum visit. The numerator should be the # of people of this group who are still breastfeeding. This doesn't seem to be how the authors calculated these rates.

Thank you for this perspective. We have included a brief calculation of our rates below:

12/34 = 0.3529 * 100 = 35.3%
33/54 = 0.6111 * 100 = 61.1%

There is additional documentation related to this decision letter. To access the file(s), please click the link below. You may also login to the system and click the 'View Attachments' link in the Action column.

<https://www.editorialmanager.com/rehd/l.asp?i=300653&l=1SAJT6W7>
